# Clinical biomarkers for Lewy body diseases

**DOI:** 10.1186/s13578-023-01152-x

**Published:** 2023-11-14

**Authors:** Mai M. Abdelmoaty, Eugene Lu, Rana Kadry, Emma G. Foster, Shaurav Bhattarai, R. Lee Mosley, Howard E. Gendelman

**Affiliations:** 1 Department of Pharmacology and Experimental Neuroscience, College of Medicine, University of Nebraska Medical Center, Omaha, NE 68198 USA; 2 Department of Cellular and Integrative Physiology, University of Nebraska Medical Center, Omaha, NE 68198 USA

**Keywords:** Synucleinopathies, Parkinson’s disease, Parkinson’s disease with dementia, Dementia with Lewy bodies, α-synuclein, Biomarkers

## Abstract

Synucleinopathies are a group of neurodegenerative disorders characterized by pathologic aggregates of neural and glial α-synuclein (α-syn) in the form of Lewy bodies (LBs), Lewy neurites, and cytoplasmic inclusions in both neurons and glia. Two major classes of synucleinopathies are LB disease and multiple system atrophy. LB diseases include Parkinson’s disease (PD), PD with dementia, and dementia with LBs. All are increasing in prevalence. Effective diagnostics, disease-modifying therapies, and therapeutic monitoring are urgently needed. Diagnostics capable of differentiating LB diseases are based on signs and symptoms which might overlap. To date, no specific diagnostic test exists despite disease-specific pathologies. Diagnostics are aided by brain imaging and cerebrospinal fluid evaluations, but more accessible biomarkers remain in need. Mechanisms of α-syn evolution to pathologic oligomers and insoluble fibrils can provide one of a spectrum of biomarkers to link complex neural pathways to effective therapies. With these in mind, we review promising biomarkers linked to effective disease-modifying interventions.

## Introduction

Synucleinopathies are neurodegenerative diseases that share the presence of α-synuclein** (**α-syn) aggregates in neurons and glia, and are found as Lewy bodies (LBs), Lewy neurites (LNs), and neuronal and glial cytoplasmic inclusions [1]. The disease spectrum includes Parkinson’s disease (PD), PD dementia (PDD), dementia with Lewy bodies (DLB), and multiple system atrophy (MSA) [2]. The three main α-synucleinopathies are PD, DLB, and MSA [3]. The most common α-synucleinopathy is PD [4], while the others are less common, differential disease diagnoses among them are clouded. This is made ever more difficult as PD affects up to 2% of the population above 60 years of age. Yearly, 90,000 people in the United States are newly diagnosed with PD to yield a prevalence of 10 million people worldwide [5, 6]. Globally, PD-associated disability and death are rising faster than other neurological disorders. In the past quarter century, PD prevalence has doubled and continued to rise with global population aging. According to the World Health Organization (WHO), more than 8.5 million individuals were afflicted with PD. In 2019, PD has resulted in 5.8 million disability-adjusted life years (DALYs); an 81% increase since 2000 [7]. Most concerning rests in epidemiological studies which indicate that up to 40% of PD patients also have dementia in which disease incidence rates are increasing 4–6 times compared to those without PD [8–11]. At least 75% of patients who live with PD for more than 10 years will develop dementia [12, 13].

Lewy body dementia (LBD) describes neurodegenerative disorders characterized by the pathological aggregation of α-syn into LBs in the brain [14]. Two well-known subtypes of LBD include DLB and PDD. While DLB and PDD clinical pathologies overlap, they are differentiated by the chronology of symptoms [15]. Patients diagnosed with Parkinsonism prior to the development of cognitive impairments are classified as having PDD while those who develop cognitive impairment prior to or within 1 year of Parkinsonism are classified as having DLB [16]. DLB is the second most common form of dementia after Alzheimer’s disease (AD) and is 20% of the total case numbers [17]. PDD is a second type of DLB, but both have unique disease courses [18]. The incidence and prevalence of PDD and DLB vary greatly in clinical studies and population-based cohorts [19]. In Minnesota alone, from 1991 to 2005, the incidence rate of DLB and PDD was 31.6 and 23, respectively, per 100,000 person-years [20]. Other studies reviewing LBD subtypes in the United States showed 0.02% and 4.4–5.4% amongst Floridians and all dementia cases in Medicare beneficiaries, respectively [21, 22]. A recent study showed that in the United States from 2010 to 2016, the incidence and prevalence of LBD among Medicare beneficiaries ranged from 0.18–0.21% to 0.83–0.9%, respectively. The costs of treating LBD were $18,309 for the pre-diagnosis and $29,174 and $22,814 at years 1 and 5 after diagnosis [23]. Comparisons between studies are challenging because of divergent study designs, patient populations, and disease time course.

The accurate diagnosis of these synucleinopathies and timely and cost-effective follow-ups as well as therapeutic response monitoring represent great needs. These include monitoring of treatment regimens and facilitating patients’ enrollment into clinical studies. Moreover, misdiagnosis can lead to suboptimal treatment, unnecessary care, and costly follow-ups to confirm diagnoses [24, 25]. The poor diagnostic accuracy for synucleinopathies in the absence of pathology-specific biomarkers increases the risks of confounding clinical trial inclusions and accuracies. Accurate, easily accessible, and cost-effective diagnostic biomarkers permit accurate clinical use for evaluating disease progression and disease-modifying therapies (DMTs). Optimal time windows exist during disease progression when DMTs may be most effective. Targeting key pathological hallmarks, such as α-syn misfolding and aggregation in synucleinopathies, will likely be a first step toward more effective therapies with improved and earlier interventional modalities before disease continues toward irreversible neurodegeneration [26, 27]. Therefore, biomarkers detecting disease pathology before the onset of disabling symptoms are needed.

This review is divided into three main sections reflective of the scenario occurring in the medical and pharmaceutical fields. Herein, we summarize the traditional signs and symptoms used in the clinic to establish a LB diagnosis. Next, the underlying disease mechanisms are presented, followed by prospects for improving diagnostics and treatment interventions in the future. These needs are discussed in context of biomarkers available for diagnostic and prognostic purposes. Last, we present more translational biomarkers that track disease and therapeutic responses.

## Current available diagnostics

Some neurodegenerative diseases are characterized by abnormal aggregation and accumulation of pathologically altered proteins that are specific to disease groups and are typically designated as proteinopathies. Simultaneously, they involve dysfunctions of a range of cellular mechanisms, including mitochondrial or lysosomal dysfunction, oxidative stress, and inflammation caused primarily by glial cell activation that ultimately results in neuronal degeneration [28]. These mechanisms are common for several neurodegenerative diseases and may vary with degrees of neurodegeneration.

**Fig. 1:**
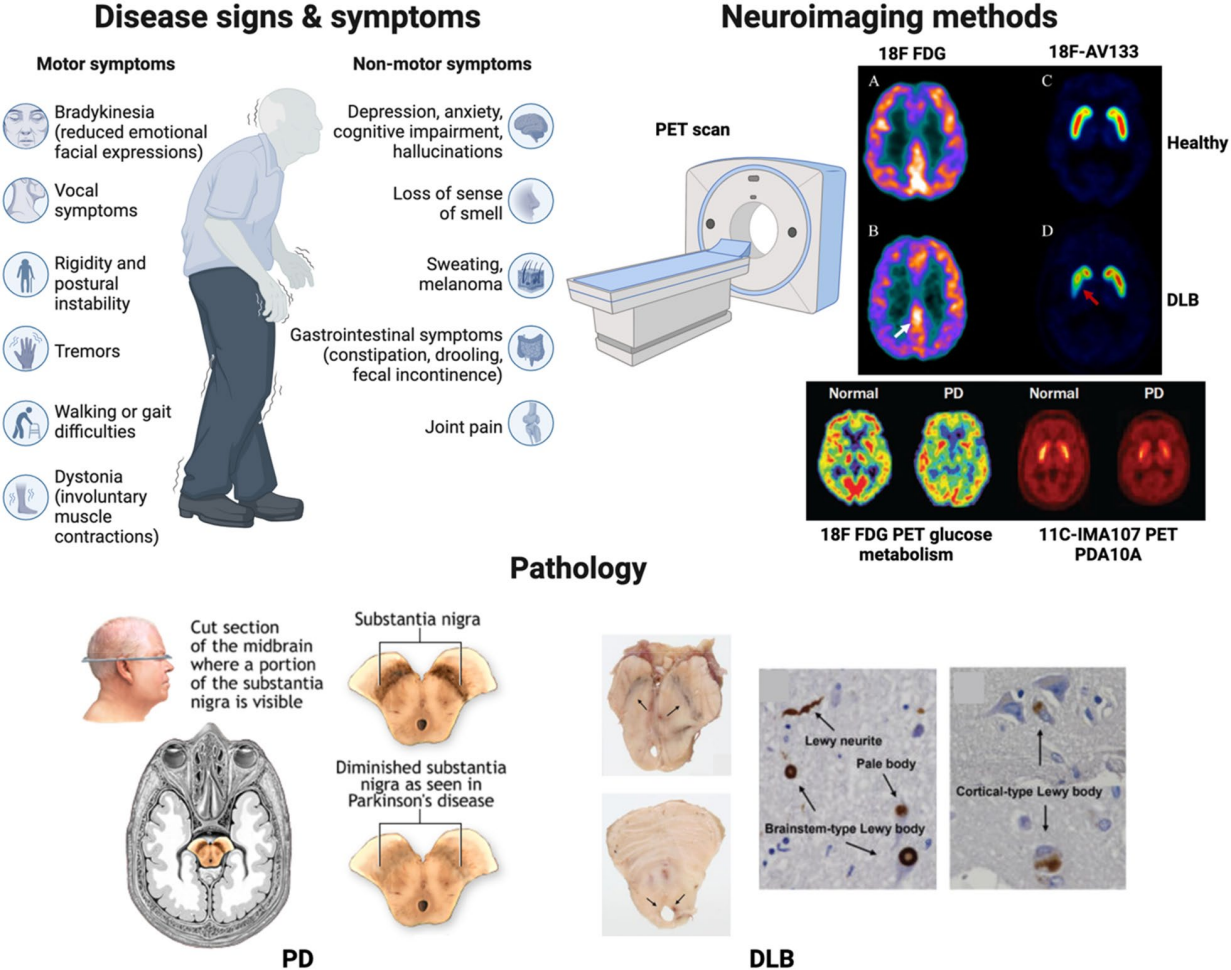
LB disease signs, symptoms, diagnostics, and disease pathobiology. Disease signs and symptoms: Motor and non-motor symptoms are both included as operative processes leading to significant disability. Motor symptoms include bradykinesia, tremors, rigidity, and postural instability. The non-motor sings include, but are not limited to, depression, anxiety, hyposmia, and constipation. Neuroimaging methods: Tomographic acquisitions for neurodegenerative diseases include positron emission tomography (PET) scanning using ^18^F-fluorodeoxyglucose (^18^F-FDG) and ^11^C-IMA107 for DLB [31] and PD [32]. Pathology: The pathologies of synucleinopathies such as PD [33] and DLB [34] show depigmentation in the midbrain substantia nigra, Lewy neurites, and Lewy bodies (black arrows). The figure was created with BioRender.com. Images taken from publications or web pages were referenced in the figure caption

Neurodegenerative diseases employ multiple categories of diagnostics such as clinical signs and symptoms, neuroimaging acquisitions, genetic markers, and biological and biochemical indicators in cerebrospinal fluid (CSF), blood, or tissues **(**Fig. 1). In addition to transcriptomic studies, other “omic” technologies such as proteomics and metabolomics, both of which study functional molecules (proteins and neurotransmitter metabolites) that are potentially involved in neurodegenerative processes, are increasingly being used [29, 30]. A main objective is to uncover a signature profile, i.e., combinations of biomarkers specific to a specific disease or a group of diseases that share common pathologies or processes.

### Clinical signs and symptoms

For all synucleinopathies, α-syn accumulation in LBs with associated loss of substantia nigra pars compacta (SNpc) dopaminergic neurons and dopamine neurotransmitter heralds motor and non-motor symptoms. These are significant sources of each patient’s clinical disabilities. Cardinal motor symptoms of PD include bradykinesia, tremors, rigidity, and postural instability. Bradykinesia presents as slowness of movement interrupted by halts in movement [35]. Another indicator for PD is a unilateral tremor that occurs while the afflicted limb is at rest and can manifest in legs, lips, and chin [36]. Rigidity in the flexion and extension of limbs is another characteristic of PD. Posture instability is due to the inability of maintaining equilibrium in a static state, such as sitting or standing, or instability in the transition from static state to a moving state [37]. Two forms of PD can be distinguished clinically with tremor or axial form depending on the predominance of symptoms. Motor symptoms begin insidiously and progress gradually over time [38]. In addition, the spectrum of symptoms for PD has expanded to non-motor symptoms, which often precede the onset of motor symptoms by 5–10 years [39]. These non-motor symptoms can range from mood changes to alterations in sleep habits [40]. PD patients have also experienced olfactory dysfunctions [41]. In a longitudinal study, PD patients with higher United Parkinson’s Disease Rating Scale (UPDRS) scores showed higher levels of olfactory dysfunction compared to PD patients who had lower UPDRS scores [42]. Rapid-eye-movement (REM) sleep behavior disorder (RBD) has also been seen as a pre-clinical indicator for PD [43]. Constipation is also associated with PD, which underscores the importance of the gut-brain axis due to the hypothesis that PD etiology may originate in the gut and migrate to the brain [44].

One of the most common disabling non-motor features in PD is dementia [45]. PDD and DLB are two disorders which encompass Parkinsonian-related dementias. The temporal onset of dementia is a major discriminating factor between PDD and DLB and it is termed the “1-year rule”. In DLB, onset of cognitive decline precedes presentation of PD symptoms by 1 year or less. While in PDD, cognitive decline typically develops within 1 year of PD diagnosis [46]. Thus, the temporal onset of dementia and Parkinsonism for PD, PDD, and DLB could provide beneficial importance for the differential diagnoses of those disorders (Table 1**)**. Additional clinical factors have been associated with PDD such as older age of PD onset, male sex preponderance, hallucinations, increased severity of motor symptoms and bradykinesia, akinetic-dominant Parkinsonism, axial impairment, and depression [13, 47, 48]. Further, RBD, posterior-cortical dysfunction, cardiovascular autonomic dysfunction, color discrimination ability, and gait dysfunction are strong predictors of development of PDD in PD patients [49, 50], whereas in DLB, cognitive decline is not accompanied by PD symptoms and typically precedes PD diagnosis [46]. The cardinal features of DLB include dementia, fluctuating cognition, visual hallucinations, RBD, and Parkinsonism [16]. Motor symptoms may be absent in up to 25% of autopsy confirmed DLB patients [51]. While DLB patients share many symptoms with PD, cognitive impairment including deficits in attention, executive function, and visuospatial ability, precede the onset of motor symptoms [18, 52]. A consensus for differentiating LB diseases were developed from four studies of a DLB consortium from 1996 to 2017 [16], and established a set of criteria for diagnosing PDD and DLB [53]. Dementia associated with DLB or PDD is defined as a progressive cognitive impairment that interferes with daily life activities and normal work-related and social occupations [54]. Screening patients for dementia should be performed according to the Mini Mental State Examination (MMSE), Montreal Cognitive Assessment (MoCA), and neurophysiological assessments and combined for initial diagnosis [55]. Reviews elsewhere provide criteria for PDD diagnosis [56, 57], and revised criteria according to DLB Consortium for DLB diagnosis [16, 58].

**Table 1:** Onset of Parkinsonism and dementia in PD, PDD, and DLB

Clinical feature	PD	PDD	DLB
Parkinsonism	Earlier	Earlier	Absent or later
Dementia	Absent	Later	Earlier

### Neuroimaging

Intensive research using neuroimaging techniques allowed assessment of structural and functional alterations of neurodegenerative patients and enhanced the criteria for differential diagnoses for DLB, PD, PDD, and AD [59]. In PD, different imaging techniques can verify the validity of a PD diagnosis from the motor and non-motor symptoms. Magnetic resonance imaging (MRI) scans provide deeper insight into the progression of PD and differentiate PD cases from other neurodegenerative diseases. The onset of PD leads not only to neuronal changes within the brain, but also large-scale physical changes such as atrophy in many cortical and subcortical areas along with overall decreases in brain volume [60] and increased frontal lobe volumes [61]. Structural MRI, including T1- and T2-weighted imaging, is used to study patterns of brain atrophy as an estimate of regional neurodegeneration. MRI measures of gray matter volumes and cortical thickness often exhibit low intra-individual variability over time, therefore small differences in atrophy rates can be detected in longitudinal clinical trials [62]. With measurement of other structural characteristics associated with PD, MRI provides an instrument capable of capturing small spatial differences within strictly defined areas of the brain. Moreover, thin section T2 and proton density spin echo images have been able to discriminate between PD and MSA with 88% sensitivity and 89% specificity [63]. Another MIR focus is iron mapping. Iron content is known to be regionally increased in the substantia nigra of PD patients compared to normal healthy controls [64]. This gives us the ability to create a reference for diseased brains compared to normal brains. A meta-analysis study showed that MRI measures of iron content in PD patients across multiple studies provided a reliable marker for PD that correlated with the severity of motor symptoms [65]. Unfortunately, information about loss of cell populations or cellular structures is not yet possible with MRI. However, for several neurodegenerative diseases, the blood–brain barrier (BBB) integrity is disrupted and can be evaluated by certain MRI techniques, including dynamic contrast-enhanced and dynamic susceptibility contrast MRI, which depend on a more permeable BBB and leakage of gadolinium-based contrast agents into the brain [66].

Many avenues have assessed specific synaptic connections between the dopaminergic neurons for PD diagnosis and progression [67]. Loss of the dopamine transporter (DAT) in the substantia nigra has gained much attention as a possible biomarker for PD. Positron emission tomography (PET) imaging can be used to determine the density of dopaminergic nerve terminals in the basal ganglia which is typically reduced in PD, MSA, progressive supranuclear palsy (PSP), and corticobasal degeneration [68]. Recently, PET ligands binding to the synaptic vesicle protein 2A (SV2A) were found to detect regionally decreased synaptic density in PD and AD [69, 70]. ^18^F-FDG-PET has been used for diagnostic workup of several neurodegenerative diseases, in part, to certain patterns of regional hypometabolism. For example, frontal and anterior temporal hypometabolism are typically observed in the behavioral variant of frontotemporal dementia (FTD) [71]. ^11^C-dihydrotetrabenazine (^11^C-DTBZ) is another PET ligand for vesicular monoamine type 2 transporter (VMAT2) within presynaptic dopaminergic neurons. ^11^C-DTBZ PET imaging with correlational tractography showed diminished nigrostriatal tract integrity with lower ^11^C-DTBZ distribution in PD patients, which suggested nigrostriatal axonal dysfunction [72]. These imaging methods are often used in studies to evaluate the potential neuroprotective effects of treatments on dopaminergic neurons. Another popular imaging probe is ^123^I-ioflupane detected by single-photon emission computed tomography (SPECT). The combination is known as dopamine transporter scan (DaTscan) [32]. This type of imaging allows better differentiation between PD and other types of Parkinsonism. Alternative nuclear medicine techniques have also been applied along with subsequent radiolabeled tracers. One such example is the ^18^F-FE-PE2I PET imaging, which has proven comparable sensitivity to mainstream ^123^I-ioflupane SPECT imaging [73, 74].

Another promising MRI method is diffusion tensor imaging (DTI) which is used for measuring fractional anisotropy and microstructural indices of brain white matter in different neurodegenerative diseases [75]. Lower than normal fractional anisotropy and higher than normal diffusivity is associated with loss of microstructural integrity and neurodegeneration. Previous DTI studies in PD demonstrated abnormal fractional anisotropy in multiple white matter regions as well as in the dopaminergic nuclei [76]. DTI has also shown promise in discerning PD patients from healthy subjects. In a meta-analysis of 39 studies, mean diffusivity and fractional anisotropy found differences in the substantia nigra, corpus callosum, and cingulate and temporal cortices of PD patients [77]. In addition, DTI has been used to differentiate PD patients from PDD patients by assessing microstructural changes in the nucleus basalis of Meynert (NBM). The NBM of PDD patients revealed increased mean diffusivity and reduced gray matter volume compared to PD patients without cognitive impairment. This microstructural change in the NBM was shown to precede gray matter volumetric loss suggesting an early biomarker for PDD [78]. Furthermore, in PDD, the combination of DTI with resting state functional MRI (fMRI) showed diminished functional connectivity of the posterior cingulate-right medial temporal lobe as well as microstructural damage to the left hippocampus [79]. This suggests that using a combination of imaging techniques could provide predictive markers of PDD. Other imaging studies have found that cortical thinning of the frontal, right precentral, and anterior cingulate cortex in combination with gray matter atrophy are predictive of cognitive decline in PD [80, 81].

^123^Iodine-meta-iodobenzylguanidine (^123^I-MIBG) myocardial scintigraphy is another imaging tool used initially to assess sympathetic denervation, density, and function in organs richly innervated by sympathetic nervous system such as the postganglionic presynaptic of the cardiac sympathetic nerve endings. The radiolabeled ^123^I-MIBG (a norepinephrine analogue) is taken up by postganglionic postsynaptic nerve endings and its uptake was found to be correlated to adrenergic innervation and integrity of substantia nigra neurons [82]. ^123^I-MIBG was found to differentiate between DLB and AD and predict the prognosis of DLB, due to the correlation of postganglionic neurons associated with DLB. The technique was included in DLB consortium criteria as an indicative biomarker due to the high sensitivity to discriminate between DLB and AD via reduction of cardiac uptake of ^123^I-MIBG by DLB patients, but not AD patients [83].

## Disease pathobiology for diagnostic/therapeutic development

### Genetics

Research performed in the past two decades identified multiple autosomal recessive and dominant genes associated with familial PD. Duplicate or triplicate mutations in α-syn gene (*SNCA*) cause dominant inherited forms of PD [84, 85]. Additionally, genome-wide association studies (GWAS) have uncovered variations in at least two of the familial PD genes (*SNCA* and leucine-rich repeat kinase 2; *LRRK2*). These have proven to be significant risk factors for sporadic PD [86–89]. Missense mutations in *SNCA* were identified in familial PD (A53T, A30P, E46K, and H50Q) [90, 91] as well as in sporadic PD (A18T and A29S) [92]. Six mutations in *LRRK2* were identified as disease-causing: G2019S, R1441C/G/H, Y1699C, and I2020T in familial PD [93, 94]. G2019S and R1441C mutations are responsible for up to 30% of familial PD in select populations, and up to 10% and 2.5%, respectively, in sporadic PD [95, 96]. Moreover, studies identified associations between PD and both *PARK*-designated genes (*SNCA, PRKN, UCHL1, PINK1, DJ-1, LRRK2, ATP13A2, GIGYF2, HTRA2, PLA2G6, FBX07, VPS35, EIF4G1, DNAJC6, SYNJ1, DNAJC13, and VPS13C*) and non-*PARK*-designated genes (*BST1, CCDCC2/HIP1R, DGKQ/GAK, GBAMAPT, MCCC1/LAMP3, STK39, SYT11/ RAB25, GAK, MAPT, GBA, NAT2, INOS2A, GAK, HLA-DRA,* and *APOE*) [97].

Associations between impaired protein and mitochondrial homeostasis and the development and progression of PD were shown. This notably includes oxidative stress acting as an important link between the pathogenic events. In addition to abnormal protein overproduction and aggregation, impaired degradation pathways, such as lysosomal dysfunctions and autophagy contribute to PD [98, 99]. Autophagy serves to remove aggregated misfolded proteins and dysfunctional organelles to clear pathologic components and prevent toxicity and subsequent cell death. Multiple studies suggest that aggregation of α-syn is consequent to dysfunction of the autophagic-lysosomal system. α-Syn also affects mitochondrial, lysosomal, and autophagic functions [100–102]. Dopaminergic neurons are metabolically active and need high mitochondrial energy demand, and therefore are exposed to insufficient clearance of damaged mitochondria [103]. Accumulation of defective mitochondria will increase levels of reactive oxygen species (ROS), which damage surrounding healthy mitochondria and accelerate disease progression. Overall, elevated α-syn concentrations from either overproduction or reduced clearance, lead to α-syn aggregations and neurotoxicity. Therefore, lowering α-syn levels reduces oligomerization, aggregation, and deposition into LBs. This can result in a beneficial disease-modifying effect for synucleinopathies.

Recently, GWAS of a cohort of 2,591 patients diagnosed with DLB and 4,027 healthy controls from across 17 European and 27 North American sites resulted in identification of the highest independent five loci risks (*SNCA-AS1, GBA, APOE, B1N1,* and *TMEM 175*) [104]. GWAS and co-localization identified two loci risks, *SNCA* and *SNCA* antisense RNA 1 (*SNCA-AS1*), a non-coding RNA playing a role in regulating expression of α-syn. Transcriptomic studies showed overexpression of both genes. These works illustrated the impact on synaptogenesis and the role of *SNCA-AS1* in cellular senescence and PD-related pathologies [104, 105]. Due to the overlap between α-syn, tau, and amyloid beta (Aβ) pathologies in DLB and PDD, an opportunity presents to compare genotypes with prognostic and therapeutic values between AD, PD, PDD, and DLB. Glucocerebrosidase (*GBA)* mutations are critical loci in DLB pathogenesis, and *GBA* gene variants were the first genetic risk factor identified for PD [106]. *GBA* encodes for the lysosomal hydrolase enzyme β-glucocerebrosidase (GCase) which catalyzes the hydrolysis of glucocerebroside to ceremide and glucose. This leads to increased GCase misfolding, endoplasmic reticulum (ER) trapping, and induction of both ER stress and ubiquitin proteasome (UPS) systems. This triggers ER associated degradation (ERAD) and unfolded protein response (UPR). Eventually, sustained activation of ERAD and UPR will increase apoptosis. The presence of misfolded GCase in lysosomes leads to accumulation and aggregation of α-syn. This results in decreasing α-syn degradation through chaperon mediated autophagy (CMA). *GBA* mutations also affect mitochondrial dysfunction by increasing ROS and decreasing ATP production and oxygen consumption [107].

Apolipoprotein E glycoprotein (APOE) maintains cholesterol hemostasis through facilitating transfer of phospholipids and cholesterol amongst cells. In the central nervous system (CNS), APOE is produced by astrocytes more than by microglia. This explains APOE upregulation in neurodegenerative diseases and its association with neuroinflammation. The process proceeds through activation of astrocytes and microglia. APOE lipidation mediates Aβ clearance through the astrocyte ATP-binding cassette A1 (ABCA1) cascade and inhibits Aβ plaque formation. One potential mechanism of Aβ plaque formation involves APOE protein overexpression, susceptibility to mutation, and accumulation of acids and triglycerides, which form complexes that bind Aβ aggregates to form Aβ plaques. Different APOE isoforms (APOE1, 2, 3, and 4) are present. APOE4 is associated with DLB and PDD pathologies [108]. In a cohort study of 100 PD patients, associations were made between APOE4 allele carriers and a higher risk of early dementia [109]. It is unknown whether APOE4 contributes directly to or is dependent on Aβ pathology that leads to α-syn accumulations. A prior study of APOE4 in an adeno-associated virus (AAV)-α-syn-overexpressing mouse model demonstrated increased neurodegenerative and behavior deficits, and neuroinflammatory responses which were independent from Aβ pathology compared to mice which expressed other APOE variants [108]. These results were supported by human postmortem brain examinations obtained from DLB with AD pathology. These examinations showed increased α-syn pathology in APOE4 carriers compared to non-carriers [108]. Certainly, additional studies are warranted to confirm the role of APOE and interacting proteins in neurodegenerative processes. Recent investigations demonstrate that the bridging integrator 1 protein (BIN1) is linked to endosomal trafficking leading to tau pathology. Meta-analysis shows positive association between BIN1 and the APOE4 carrier allele [110, 111].

Defective lysosomal acidification is associated with several neurodegenerative disorders [112]. The optimization of pH is determined by influx and efflux proton pump dynamics. Vacuolar-type H^+^-ATPase (V-ATPase) is a known proton pump with influx activity [113], while transmembrane protein 175 (TMEM175) is an endolysosomal potassium channel that is a selective permeable efflux H^+^ proton pump when the luminal domain faces an acidic pH environment, and functionally balances the effect of V-ATPase. The role of TMEM175 in PD pathology is somewhat controversial as to whether its depletion or overexpression is pathological in PD. Studies showed that TMEM175 deficiency in PD is associated with increased loss of dopaminergic neurons and deposition of α-syn aggregates [114, 115]. However, other studies showed that TMEM175 activity is correlated with Bcl-2 apoptosis regulator factor which plays a role in mitophagy. Upon binding to Bcl-2, TMEM175 is activated and induces ROS in a TMEM175-ROS positive feedback loop manner and exacerbates dopaminergic neurons loss and motor dysfunction in PD animal models [116]. Several studies investigated which alleles of TMEM175 are associated with synucleinopathies. As in the GWAS mentioned earlier, TMEM175 rs6599388-T was shown to be a risk allele for DLB [104]. A recent genomic analysis study in Italy included 400 PD patients and 300 healthy controls, showed a strong correlation between TMEM175 variant rs2290402 allele and PD pathology [117]. Taken together, more studies are required to illustrate how mutations and variants of TMEM175 and pH changes, affect synucleinopathy etiology and progression as well as explain the dichotomous discrepancies in TMEM175 expression and PD pathology.

### Misfolded proteins

One mechanism common to several neurodegenerative diseases is the overproduction of aberrant proteins that has the potential to be harnessed for diagnostic benefit. The proteins linked to disease evolve, aggregate, and then accumulate as intra- and extra-cellular bodies. Collectively, they facilitate neuronal death in afflicted brain locations [118]. In each of the synucleinopathies, misfolded and accumulated α-syn proteins, presenting as fibrils and LBs, represent characteristic hallmarks of dopaminergic neurodegeneration as it occurs in the SNpc [119, 120]. Encoded by the *SNCA* gene, α-syn is a presynaptic protein in neurons. α-Syn is a small acidic protein expressed in the CNS, peripheral nervous system (PNS), blood, and other tissues [121]. α-Syn is natively unfolded monomer, however it is found naturally, in large part, as a folded tetramer of 58 KDa with little or no amyloid-like aggregation potential [122]. Monomer and tetramer forms exist together, but an unbalanced tetramer:monomer ratio leads to the predominance of pro-aggregating forms. α-Syn was found to have three main regions; each region has different molecular and biological properties [123]. The N-terminus, amino acid residues 1–60, is characterized by amphipathic repetitions which form an α-helix structure. This region of the protein controls the interaction of α-syn to the membranes [124]. The non-Aβ component (NAC region), amino acid residues 61–95, is the most aggregation-prone region. The C-terminus, amino acid residues 96–140, is involved in Ca^2+^ binding and chaperone-like activity [125]. It was found that binding of Ca^2+^ to the C terminus of α-syn also regulates binding to synaptic membranes [126]. The exact physiological functions of α-syn are not fully known, but most likely play a role in synaptic vesicle release. This is reflected by α-syn localization to the nerve terminal where neurotransmitter release is inhibited with α-syn blockage or deletion. In addition, α-syn aggregates localize more in brain stem and substantia nigra [127, 128]. In synucleinopathies, α-syn has a pathological β-sheet conformation that allows monomers to form oligomers and amyloid fibrils. α-Syn aggregates into LBs which localize in the neuron soma or into LNs in axons [123, 126]. While LBs are themselves not toxic, aggregates of α-syn are passed between neurons facilitating disease spread amongst adjacent brain regions [127]. During disease, α-syn filaments accumulate in amygdala and striatum forming LNs which inhibit axonal transport and reduce neuronal function and survival [129]. The localization of prion-like aggregates of α-syn is different among DLB and PD. In PD, α-syn aggregates localize in the brain stem and substantia nigra, while in DLB α-syn aggregates are diffuse throughout the brain [128]. In PD, LBs and LNs are in the mesencephalon, while in the cerebral cortex of DLB brain tissues [130].

DLB and PDD are heterogeneous disorders with overlapping clinical features amongst PD and AD, which complicate distinguishing between PDD and DLB as they share considerable clinical features [51]. Differences are seen solely in postmortem analyses and in part for imaging. Investigations in PD patients show that LB pathology is restricted to the brain stem and limbic subregions, while in PDD and DLB, LB pathology extends to the neocortex [131]. Striatal α-syn is detected in PD and PDD, but less in DLB. The dopaminergic loss in SN is higher in PD and PDD than in DLB. In contrast to DLB, more LB accumulates in neocortical and limbic regions in PDD where the temporal lobe and CA2 region of hippocampus are disease targets for PDD [132]. More postmortem studies to confirm differences in α-syn aggregation between the synucleinopathies remain in need. Postmortem studies also show that striatal Aβ loads are equivalent between AD and DLB. Aβ levels are higher in DLB than in PDD [18, 133]. Higher Aβ burdens are found in cortical and subcortical regions in postmortem samples of DLB patients than in PDD [18, 132, 133]. Hyperphosphorylated tau and Aβ, additional AD pathological hallmarks, are known to contribute to cognitive decline in PDD and DLB. DLB patients show advanced AD pathology compared to PDD patients, while patients with AD pathology are less likely to present with DLB clinical symptoms, even if diffuse LBs exist in the cortex [134]. Additionally, DLB- and PDD-associated cholinergic neuronal loss correlate better with cognitive decline. These findings affirm that cholinesterase inhibitors can provide improvement in cognitive function in DLB and PDD [135–137].

### Immunity

Neuroinflammation in neurodegenerative disorders was reported in the early 1980’s, and has been confirmed by several subsequent studies showing the link between neuroinflammation and PD pathogenesis which includes increased pro-inflammatory cytokines in blood and CNS [138, 139]. Interleukin-1beta (IL-1β), an inflammatory cytokine, is part of the larger IL-1 family and plays an important role in controlling many innate immune responses [140]. IL-1β was found to increase dopaminergic neuron damage within substantia nigra in an adenoviral IL-1β expression vector model [141]. In a clinical trial, levels of serum IL-1β, IL-6, and IL-1 receptor antagonist (IL-1Ra) were significantly elevated in PD patients [142]. Additionally, in another study, IL-1β was found to be predictive of disease progression in PD [143]. Serum IL-6 has also been found to have a negative correlation with the Activities of Daily Living (ADL) scale, which contributes to the severity of disease [138]. Another pro-inflammatory cytokine of interest in PD pathogenesis is tumor necrosis factor-alpha (TNF-α) which is a crucial component of microglia-derived inflammatory responses. Serum TNF-α has been found to be increased in PD patients compared to controls and is positively correlated with UPDRS Part III scores [144]. In another study TNF-α levels in tears of PD patients were higher compared to healthy controls [145]. C-C motif chemokine ligand 5 (CCL5) has also been found to be associated with the severity and length of PD [146]. One area of interest is the association between gut inflammation and PD as many PD patients present intestinal maladies either prior to or during motor dysfunction. Congruent with those observations were increased expression of *TNF-α,* interferon-gamma *(IFN-γ), IL-6,* and *IL-1β* genes in ascending colons of PD patients compared to age matched healthy controls [147].

Several reports documented the activation of both innate and adaptive immune systems in synucleinopathies [148–150]. Evidence implicates misfolded α-syn itself as a main trigger of immune responses and a potent inducer of an inflammatory environment, which leads to neurodegeneration. In PD, α-syn aggregation occurs in the neurons of the SNpc in the CNS and those in the PNS [149]. Microglia are the resident immune cells in the brain and function as the primary contributor to innate immunity in the CNS [151]. Activated microglia surrounding degenerative dopaminergic neurons in the SNpc were found in PD brains [152]. The degree of microglial activation was shown by CD68 and major histocompatibility complex class II (MHC-II) staining [153]. Activation of innate immunity leads to production of pro-inflammatory cytokines. These include TNF-α, IL-1β, IL-6, and IFN-γ as well as production of chemokines and activation of the complement system. Although microglia are morphologically and functionally like circulating monocytes and tissue macrophages, they originate from a different lineage in the yolk sac and migrate into the brain during early development [154, 155]. A mixture of resident brain microglia and infiltrating peripheral monocytes exert different effects in PD pathogenesis. Cytokine and chemokine production are also upregulated by peripheral blood mononuclear cells in PD patients. Levels of chemokine CC-motif ligand 3 (CCL3), CCL5, IFN-γ, monocyte chemoattractant protein-1 (MCP-1 or CCL2), IL-1β, IL-8, and TNF-α in PBMCs at baseline or stimulated with lipopolysaccharide were found to correlate with motor function assessed by UPDRS Part III and Hoehn and Yahr (H&Y) stage [156]. Additionally, complement C1q was found to be associated with activated microglia surrounding degenerating neurons [157], with C3d, C4d, C7, and C9 components found co-localized with α-syn aggregations and degenerating neurons in PD autopsies [158]. Moreover, microglia can phagocytose extracellular aggregated α-syn from their environment and target it to light chain 3B (LC3B) immunoreactive autophagosomes for degradation, leading to induction of downstream nuclear factor kappa-light-chain-enhancer of activated B cells (NF-κB)-dependent signaling cascades including those facilitating chemokine production [159]. Oxidative stress and upregulation of ROS were also observed in response to phagocytosis of aggregated α-syn in rat primary microglia [160]. Although microglia treated with aggregated α-syn increased MHC-II expression and antigen processing, a robust MHC-II-dependent cytokine response to aggregated α-syn was only induced in primary microglia by co-culturing them with T cells [161]. Furthermore, pathway analysis in two independent GWAS reports uncovered significant associations between PD diagnosis and SNPs in pathways encoding cytokine signaling and regulation of leukocyte/lymphocyte activity [162], and indicated that modified α-syn led to microglial or monocytic activation with production of pro-inflammatory cytokines. This shows the interplay between innate and adaptive immunity in synucleinopathies. In parallel, distribution of toll-like receptor (TLR) is affected in response to α-syn. TLR-2 was found to be upregulated, while TLR-3 and TLR-7 were downregulated in microglia pretreated with wild-type oligomeric α-syn [163].

The involvement of adaptive immunity in PD pathogenesis is explicit with certain evidence of the ability of modified α-syn forms to modulate adaptive responses in PD animal models. Several studies showed increased numbers of T cells in both α-syn and toxin animal models of PD with their involvement in the neurodegeneration [164–166]. In the 1-methyl-4-phenyl-1,2,3,6-tetrahydropyridine (MPTP) model of PD, adoptive transfer of T cells from mice immunized to the nitrated C-terminus of α-syn to mice administered MPTP leads to increased neurodegeneration [167]. In addition, with the polarization of T cells responsive to the nitrated C-terminus of α-syn to the Th1, Th2, and Th17 subtypes before adoptive transfer, both pro-inflammatory Th1 and Th17 cells were found to increase neurodegeneration in response to MPTP. Th17 cells showed a greater toxic effect than Th1 cells, while Th2 cells had no effect [168]. Similarly, in passive transfer studies into Rag1^−/−^ mice, CD4^+^ T cells acted in a FasL-dependent, IFN-γ-independent manner to mediate MPTP neurotoxicity. In mice treated with MPTP, dopaminergic neurodegeneration was attenuated in CD4^−/−^ animals, while neurodegeneration was unaffected in CD8a^−/−^ animals [169]. In addition, overexpression of α-syn in AAV constructs in rat brains leads to dopaminergic neuronal loss with increased infiltration of CD4^+^ and CD8^+^ T cells within the substantia nigra 8 weeks post-injection [170].

**Fig. 2:**
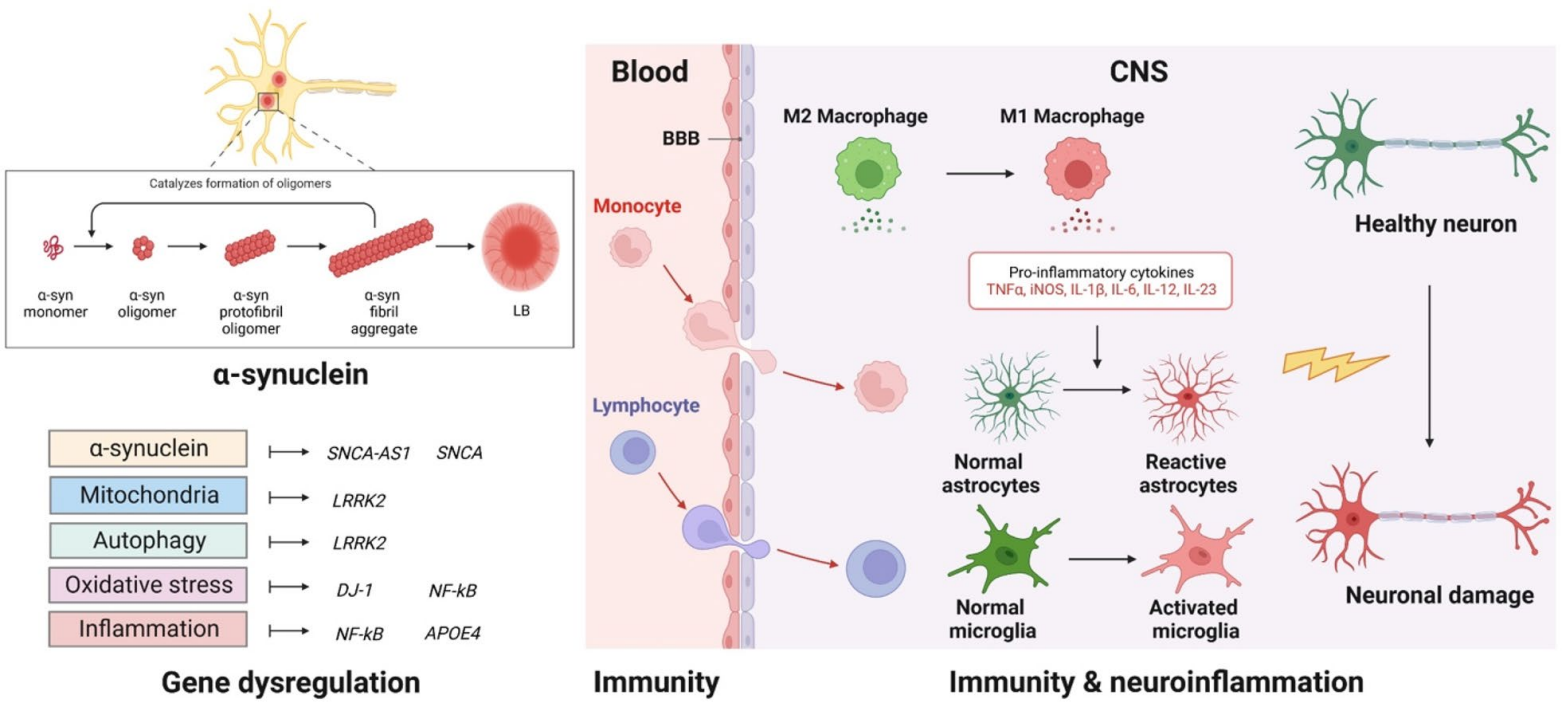
Pathogenesis of synucleinopathies. LB is mainly formed in neurons, and it is composed of misfolded, fibrillar α-syn (α-synuclein). Different genes are associated with synucleinopathies, mutations of these genes induce α-syn aggregation, mitochondrial dysfunction, autophagy dysregulation, oxidative stress, and inflammation (Gene dysregulation). Immune cells in the periphery contribute to the pathogenesis of synucleinopathies. Innate and adaptive immune cells (monocytes and lymphocytes, respectively) migrate into the brain. Activated macrophages release pro-inflammatory cytokines (Immunity). This results in generalized microglia and astrocyte activation which leads to neuronal damage (Immunity and neuroinflammation). Abbreviations: LB; Lewy body, BBB: blood–brain barrier, CNS: central nervous system. The figure was created with BioRender.com

Two subsets of functional T cells include effector T cells (Teffs) and regulatory T cells (Tregs). Functions of both subsets are maintained during homeostatic conditions to balance defense against infectious or neoplastic diseases (Teffs) and maintenance of immunological tolerance with control of overactive immune responses (Tregs). However, in neurodegenerative diseases, Teffs can recognize disease-specific modified self-proteins from oxidative stress and misfolding. These are presented by α-syn proteins in synucleinopathies, and Aβ and tau proteins in AD, which can break immune tolerance with expansion of self-reactive T cells [171, 172]. In synucleinopathies, Tregs have been found to have impaired immunosuppressive functions, whereby Teffs with neurotoxic effects are uncontrolled and expanded [173–175]. Studies from our own group showed that increased Teff phenotypes are associated with worsened UPDRS Part III scores and movement disorders in PD patients [176]. In addition, our research group, along with others, demonstrated that Tregs attenuate neuroinflammation and protect dopaminergic neurons from injury and loss in MPTP animal model [177–179]. Translationally, we have shown that strategies to increase Treg numbers or functions can modulate neuronal output and motor activity with improvement of UPDRS Part III scores in PD patients [180, 181]. Thus, modulating peripheral T cells represents viable therapeutic strategies for different neurodegenerative diseases that may express various underlying mechanisms for which these strategies are operative. Different underlying disease mechanisms contributing to synucleinopathies are summarized below (Fig. 2**)**.

## Biomarkers

**Fig. 3:**
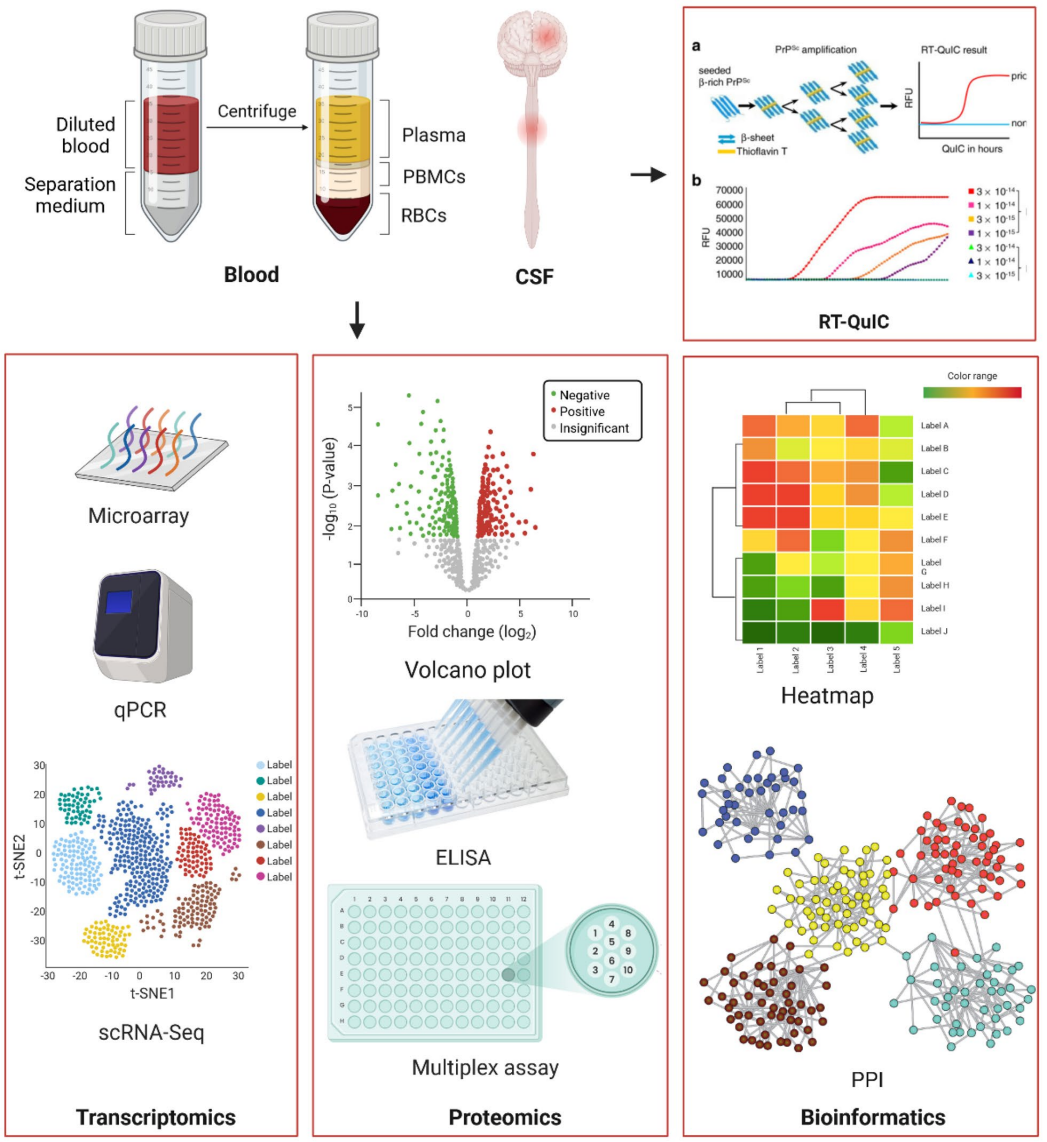
Biomarkers present or in development for neurodegenerative disorders. CSF and blood are the most common sources for samples collected for biomarker studies in neurodegenerative diseases. Samples collected from CSF and blood can be used for detecting misfolded protein by real-time quaking-induced conversion (RT-QuIC) (image [187]). Expression of genes dysregulated in neurodegenerative diseases can be assessed by different techniques such as microarrays, real-time polymerase chain reaction (qPCR), and single-cell RNA-sequencing (scRNA-seq) (Transcriptomics). Levels of proteins dysregulated in neurodegenerative diseases can be assessed by different techniques such as proteomic analysis, enzyme-linked immunosorbent assay (ELISA) (image [188]), and multiplex assay (image [189]) (Proteomics). Data from “omics” studies are processed by different bioinformatics tools generating heatmaps of altered signaling pathways, genes, and/or proteins as well as protein–protein interaction (PPI) networks (image [183]) (Bioinformatics). The figure was created with BioRender.com. Images taken from publications or web pages were referenced in the figure caption

Biomarkers are measurable indicators that serve to describe normal biological processes, pathological processes, and pharmacological responses to therapeutic interventions. The main goal of using biomarkers for neurodegenerative disorders is the improvement of clinical diagnosis which increases the accuracy of differential diagnosis between different neurodegenerative diseases. For early diagnosis, the ideal biomarker should be sensitive and specific for early disease changes that discriminate between disease state and changes due to normal aging. In addition, biomarkers help in estimating disease stage and progression as well as reflecting therapeutic responses as either change in diagnostic biomarkers or responses from therapy [118]. The revelation of new biomarkers that signal positive therapeutic effects not normally assessed in diagnostic biomarkers, could import beneficial information for clinical studies. Indeed, therapeutic biomarkers assessed as products from DMTs in early phase 1 and 2 studies are generally more likely to show clinical effects in subsequent large-scale trials [182]. In addition, development of pharmacodynamic biomarkers that can identify relevant drug targets in vivo are crucial [25]. Currently, new techniques that incorporate transcriptomics, proteomics, and metabolomics, and can be comprehensively analyzed by bioinformatics are able to uncover unique candidates for biomarker analysis and validation (Fig. 3). Neurodegenerative diseases are characterized by the interaction of multiple molecular pathways that can best be evaluated from body fluids such as CSF and blood. CSF is a most useful biological fluid as it directly reflects biochemical processes and changes within the CNS and enables early pre-clinical diagnosis when CNS biomarkers are revealed [69]. However, monitoring biomarkers for neurodegenerative diseases would be more advantageous with a more accessible and less invasive option such as blood, which communicates with the brain via the hematoencephalic barrier, lymphatic vessels, and glymphatic system [118]. Nonetheless, analyzing blood has some limitations such as whether low levels of CNS biomarkers would be even lower in the blood and become undetectable in the periphery due to the substantial analyte dilution effect from the CSF: blood volume ratio. Another limitation is a biomarker that is not specific to the CNS and co-expressed in peripheral tissues, the contribution of the CNS could be potentially lost to the higher levels associated outside the CNS. A third limitation is possible analytical interference of blood proteins such as albumins, globulins, and transferrin [118]. Therefore, measuring biomarkers of neurodegenerative diseases in the blood would require sensitive and specific tests that are not confounded by blood or blood components. Some biomarkers have been established and are being used in clinical practice (such as α-syn, Aβ_1-42_, and tau) [30, 183, 184], while other biomarkers such as LRRK2, heme oxygenase-1 (HMOX1), TLR2, autophagy related 7 (ATG7), and GBA [185, 186] are undergoing methodological and analytical standardization, and yet confirmation of other biomarkers are expected in the future.

### LB disease-aggregated protein: α-syn

α-Syn oligomers have been found within exosomes of PD patients [190], which were upregulated in plasma exosomes from PD patients [191]. Exosomal transport and reuptake is hypothesized to be a mechanism of transferring toxic α-syn species between neurons in different synucleinopathies [192]. Thus, transmission of pathogenic oligomeric forms of α-syn from neurons to microglia is entirely plausible. Several neurodegenerative biomarkers have recently emerged. These biomarkers, which reflect types of accumulated pathological proteins, include α-syn in PD and other synuclein aggregation disorders. Several studies for biomarker identification and validation focused on the measurement of total α-syn species in CSF and blood [191, 193, 194]. In synucleinopathies, a decreased α-syn level in the CSF was observed [185, 194]. The attractiveness of α-syn as a biomarker is primarily due to its increased aggregation and accumulation in the CNS. The level of total α-syn in CSF can be used to differentiate synucleinopathies from other proteinopathies; however, it is not useful for differentiating between the different synucleinopathies, even though significantly lower concentrations have been observed in MSA than in PD and DLB [185]. Specific α-syn species, such as oligomeric α-syn, phosphorylated α-syn at residue Ser129, and pro-aggregated forms of α-syn, in CSF and blood have been considered as potential diagnostic biomarkers for synucleinopathies [184].

Real-time quaking-induced conversion (RT-QuIC) analysis can directly detect pathogenic proteins with prion-like properties and is very sensitive even in the early stages of diseases that express those type of proteins. This method has been used to detect α-syn misfolding in several different synucleinopathies [195]; however, technical complexity and cost/benefit concerns have precluded wider use of this ultrasensitive method. The concentration of oligomeric α-syn in CSF is generally higher in PD and DLB patients than in healthy controls. Moreover, higher levels of phosphorylated α-syn were observed in PD patients than in healthy controls, MSA patients, or PSP patients [30, 196]. While RT-QuIC of CSF samples are used to diagnose prion diseases, measurement of pro-aggregated α-syn has the potential to diagnose synucleinopathies in pre-symptomatic stages [197]. Using this method, α-syn aggregates in the CNS were detected in the CSF of RBD patients who later developed synucleinopathy. This technique was used to analyze isolated RBD (IRBD) individuals that also developed PD. The longitudinal study found that out of 52 IRBD patients, 47 had increased CSF α-syn compared to age-matched controls [198]. Additionally, it has been shown that RT-QuIC validated the presence of α-syn aggregates in CSF of PD and DLB patients with 92% and 95% sensitivity, respectively, and with 100% specificity compared to AD patients and healthy controls [199]. Interestingly, the properties of α-syn aggregates were different between PD/DLB and MSA patients suggesting that different conformational strains of α-syn exist as distinct species for each disease [200]. Different seeding aggregation assays show high concordance for the detection of misfolded CSF α-syn when evaluated in the same cohort, indicating high reproducibility among the assays [201]. Emerging evidence shows that α-syn seed amplification assay (SAA) of CSF has the potential to differentiate PD patients from healthy controls. In this study, 1,123 participants were enrolled from 33 participating academic neurology outpatient practices worldwide (Austria, Canada, France, Germany, Greece, Israel, Italy, Netherlands, Norway, Spain, UK, and USA) between July 7, 2010, and July 4, 2019. Sensitivity for PD was 87.7% (95% CI 84.9–90.5%), and specificity for healthy controls was 96.3% (93.4–99.2%). In addition, sensitivity of α-syn SAA in sporadic PD with typical olfactory deficit was 98.6% (96.4–99.4%) [202]. Another method that detects tandem α-syn and tau aggregates is surface-based fluorescence intensity distribution analysis (sFIDA). This technique features single-particle sensitivity through a microscopy-based readout [203] and uses linear epitopes to detect and count all subtypes of aggregated protein irrespective of higher-ordered structures, whereas other assays using structural epitopes can only determine subfractions of oligomers, fibrils, or other aggregates from a heterogeneous pool of structures [204]. A recent study showed that sFIDA could discriminate between α-syn from CSF of PD patients and DLB patients with a sensitivity of 73% and specificity of 65% [204]. Although α-syn can be reliably detected in CSF, with diminished levels in PD, DLB, and MSA, substantial overlap exists with healthy controls and other neurodegenerative diseases, thereby hindering its utility in clinical practice and trials [184].

Considering the invasive nature of CSF collection, detection of α-syn in less invasive and more easily accessible fluids and tissues would be a great step forward. Preliminary results indicate that skin biopsies, which include nerve terminals, are reliable samples for use in seeding aggregation assays to detect misfolded α-syn in PD, DLB, and MSA [205, 206]. Other potential peripheral tissues for detection of misfolded α-syn included olfactory mucosa and submandibular gland tissues. Salivary RT-QuIC has also been used to detect α-syn and showed 76% sensitivity and 94.4% specificity in differentiating patient with PD (75 subjects) from healthy controls (36 subjects) [207]. In another study, postmortem submandibular gland tissue was used to sample α-syn with RT-QuIC in 32 cases (13 PD patients, 3 incidental LBD, and 16 control cases) with 100% sensitivity and 95% specificity for PD with 100% concordance for elevated levels of pathological α-syn seeding activity in both PD and incidental LBD tissues compared to control tissues [208]. However, more data from different tissues are needed to verify the sensitivity and specificity of this ultrasensitive RT-QuIC method.

In the periphery, α-syn is largely expressed and measured in blood [209], however, the quantities in blood are strongly influenced by levels of red blood cell (RBC) contamination and hemolysis, even more than in CSF. RBCs are the major source (> 99%) of α-syn in blood and their abundance and fragility make it possible that even low RBC contamination could result in a false positive increase of α-syn in serum or plasma [210]. For this reason, levels of intracellular RBC α-syn are studied as an alternative measurement. Serum and plasma levels of total α-syn have been reported to be either higher [191], lower [194], or not significantly different [193] in PD patients compared to healthy controls. α-Syn levels in blood in light of RBC contamination, limit the utility of plasma or serum total α-syn measurement for diagnostic purposes in PD. For blood oligomeric α-syn, studies provided concordant results showing increased quantities in patients with PD, both in serum and RBCs [211–213] with remarkable diagnostic accuracy in serum (sensitivity 75% and specificity 100%) compared to controls [213], but requires confirmation in larger cohorts. Like oligomeric α-syn, plasma phosphorylated α-syn levels are higher in PD patients compared with healthy controls (AUC 0.71) [193]. In this context, RBC measurements of multiple post-translational modified forms of α-syn, such as nitrated, Tyr-125 phosphorylated, SUMOylated, and glycated species, were controversial in distinguishing PD patients from healthy controls (AUC 0.84) [214]. Although extensive evidence of α-syn measurements in CSF and blood exists, a definitive biomarker for PD has not yet been discovered due, in part to RBC contamination and overlap of α-syn forms found in CSF for different neurodegenerative disorders.

### Other disease-linked proteins: Aβ, tau, and others

Other pathologic proteins have been assessed as biomarkers for different synucleinopathies, including those associated with AD pathology (Aβ_1-42_ and tau). In a small cohort (*N* = 28) of PD patients and age-matched controls, α-syn (total and oligomeric), Aβ_1-42_, and tau (total and phosphorylated) were determined in RBCs [212]. For the first time, those studies showed that PD patients exhibit α-syn heterocomplexes composed of Aβ_1-42_ and tau in RBCs. Moreover, concentrations of α-syn-Aβ_1-42_ were increased in PD subjects compared to healthy controls, and directly correlated with disease severity and motor deficits. In addition, total-α-syn levels were decreased in PD subjects and inversely related to their motor deficits. Furthermore, increased oligomeric α-syn and phosphorylated tau (ptau) from RBCs were detected in PD patients compared to controls. This study showed that combinations of total α-syn, ptau, and α-syn-Aβ_1-42_ concentrations provided the best fitting predictive index for discriminating PD patients from controls.

PDD pathologies include aggregated α-syn and LBs in the neocortex as well as Aβ plaques and tau neurofibrillary tangles. Therefore, α-syn, Aβ_1-42_, and tau in CSF have been used in several studies as biomarkers for PDD. Whereas Aβ was found to be reduced in PDD patients, those patients were still afflicted with attention deficits, executive function losses, and more rapid cognitive decline [185, 215]. Conversely, levels of total tau (t-tau) and ptau in CSF were found to be increased in PDD and more correlated to those cognitive deficits [216, 217]. In addition, the ratio of t-tau and Aβ in CSF has been used as a predictor of PDD with a low Aβ/t-tau ratio predictive of cognitive decline [218]. Conflicting results on the association of CSF α-syn levels with cognitive impairment in PD complicates its use as a biomarker for PDD [219, 220]. Data from these studies suggest that reduced Aβ and increased tau, but not α-syn in CSF can be predictors of cognitive decline in PD.

In DLB patients, Aβ_1-42_ levels were found to be significantly decreased compared to healthy controls, however decreased levels were also observed in AD patients [221, 222]. Additionally, lower Aβ_1-42_ levels were found in CSF of AD patients compared to DLB patients, with high specificity (94%), but low sensitivity (48%) [223]. Interestingly, the Aβ_1-40_ levels at different stages of the disease were found to be different between DLB and AD [224, 225]. In DLB, the decrease in Aβ_1-40_ levels is moderate compared to controls, but is not statistically significant [226]. On the other hand, in AD, Aβ_1-40_ levels rise sharply during the prodromal stage or before the onset of dementia [227]. Thus, although no significant differences in Aβ_1-42_ levels were found between DLB and AD, the decreases in Aβ_1-40_ levels in DLB results in higher Aβ_1-42_/Aβ_1-40_ ratio in DLB compared to AD. During the prodromal stage, lower Aβ_1-42_/Aβ_1-40_ ratio in AD cases helps to distinguish DLB patients from AD. This ratio can also distinguish AD from other forms of dementia, with specificity ranging from 73 to 94.7% and specificity ranging from 78 to 100% [228, 229]. Additionally, ptau protein at threonine-181 (pTau_181_) can also be used as a biomarker for DLB diagnosis [227]. The levels of pTau_181_ are higher in AD patients than in DLB patients and can be used to differentiate the diagnosis of DLB from AD with 75–94% sensitivity and 61–94% specificity. pTau_181_ levels are specific in discriminating AD from other forms of dementia as pTau_181_ levels remain unchanged in other dementias except for AD [230, 231]. The t-tau levels in CSF of DLB patients are shown to be normal or slightly lower than AD patients [232]. However, several studies refuted this finding by showing overlap of t-tau levels between DLB and AD patients and were verified by autopsy findings [233, 234]. Thus, differential diagnosis between AD and DLB is not supported based on t-tau levels in the CSF.

In recent years, other proteins have drawn attention in attempting to differentiate DLB from AD. These include chitinase-3-like protein 1 (CHI3L1, also known as YLK-40), neurogranin (NGRN), and visinin-like protein 1 (VILIP-1). YKL-40 is a glycoprotein that is expressed by a variety of cells, including macrophages, neutrophils, and chondrocytes [235, 236]. Elevated levels of YKL-40 have been found in several neurodegenerative diseases such as AD, PD, and DLB. Levels of YLK-40 in CSF were found to be significantly higher in AD compared to the DLB patients [237, 238]. NGRN is a postsynaptic protein involved in regulating synaptic plasticity and memory formation. This protein is highly expressed in the brain, particularly in the hippocampus and cortex, and has been implicated in AD and DLB pathologies. NGRN levels are higher in AD and DLB patients than in healthy controls. Despite noticeable synaptic dysfunction in DLB, levels of NGRN are significantly higher in AD compared to DLB [237]. VILIP-1 is a member of the neuronal calcium sensor protein family and has been identified as a biomarker for calcium-mediated neuronal injury [239]. VILIP-1 plays a critical role in linking calcium-mediated neurotoxicity and AD pathological changes [240]. One study has shown that CSF levels of VILIP-1 were significantly higher in AD patients compared to healthy controls and DLB patients [241]. It should be noted that overall the sensitivity and specificity of DLB diagnosis from AD has been found to range from 72–79% to 64–76%, respectively, depending on the detection limit of the analyte and the stage of each disease [227].

### Disease-related biological/biochemical biomarkers

For the synucleinopathies, biomarkers of prime interest are those that play roles in mitochondrial dysfunction, oxidative stress, and lysosomal dysfunction. Protein deglycase (DJ-1) is a multifunctional protein involved in several cellular processes with diminished function leading to increased oxidative stress. Formerly, results of CSF concentrations of DJ-1 in neurodegenerative diseases were controversial. Using a new highly ultrasensitive Luminex ELISA, decreased CSF levels of DJ-1 were found among PD patients compared to control subjects and those afflicted with AD and MSA [242]. Another potential biomarker for PD is ubiquitin C-terminal hydrolase-L1 (UCH-L1) which participates in the degradation of abnormally modified proteins from neuronal cytoplasm. Significant decreases in UCH-L1 CSF levels were found in PD compared to PSP and MSA [30, 243]. Similarly, lysosomal hydrolase GBA, which is involved in α-syn degradation, is considered a major risk factor for PD when mutated leading to diminished ability to degrade misfolded α-syn. Decreased CSF levels of GBA in early stages of sporadic PD together with increased oligomeric α-syn/total α-syn ratios have been suggested as a combined candidate diagnostic biomarker of early PD [185]. Other potential biomarkers include low serum levels of uric acid [244], epidermal growth factor [245], and insulin-like growth factor [246] that were predictive of cognitive decline in PD, and highly predictive of cognitive decline in PDD. Thus, combining several serum analytes with neuroimaging biomarkers may provide higher accuracy in diagnosing and assessing progression of PDD.

Focus on several unique micro-ribonucleic acids (miRNAs) may also provide promising biomarkers for PD. miRNAs are single stranded chains of non-coding RNA involved in regulating the expression of different genes. The dysfunction of these miRNAs in synucleinopathies can result in problems including, but are not limited to, overexpression of α-syn [247], upregulation of LRRK2 protein [248], downregulation of DJ-1 protein [249], and dysregulation of pro-inflammatory mediators [250]. miRNAs are assumed to be tissue-specific, abundant, highly stable, and quantifiable. Their upregulation or downregulation can occur several years before the onset of PD; thus, miRNAs may serve as putative biomarkers for early stages of PD [29, 30]. For example, miR-137 and miR-124 are widely expressed in the CNS and are involved in neurogenesis, neurotransmission, morphology of synapses, inflammation, autophagy, and mitochondrial function [251]. Clinical evidence showed that serum miR-137 levels are significantly increased for PD patients compared to healthy controls, while miR-124 levels were significantly down-regulated [252]. Downregulation of miR-124 was observed in early stages of neurodegeneration, implying that its reduction may not only reflect dopamine-induced cell death, but may even contribute to the initial biological process of neurodegeneration in PD [253].

The CSF-to-plasma ratio of albumin as a reflection of the integrity of the BBB was found to be elevated in most dementia disorders independent of AD pathology represents another potential biomarker for neurodegenerative diseases [254]. Several other non-specific biomarkers candidates include markers of axonal damage and degeneration, such as the well-studied neurofilament light-chain (NFL) [118]. Measured in both blood and CSF, this biomarker reflects axonal degeneration and injury, irrespective of cause, and its levels are increased in amyotrophic lateral sclerosis (ALS), FTD, and atypical Parkinsonian disorders (PSP, MSA, and corticobasal syndrome) [255]. NFL has also been found to be suggestive of PDD development and as such have certain predictive associations with PDD. High NFL protein and heart-type fatty acid-binding protein (H-FABP), in combination with low Aβ, in CSF was found to be highly predictive of future PDD [256]. NFL levels are also increased in AD, and studies on autosomal dominant AD show that the rate of change in blood NFL increases 15 years prior to onset of symptoms [257]. Importantly, higher levels of NFL are associated with faster disease progression and higher brain atrophy rates in most neurodegenerative diseases [258, 259]. Therefore, NFL can be considered as a measure of the intensity of ongoing neurodegeneration, regardless of specific disorder. Effective DMTs can normalize NFL levels, such as in treatment of multiple sclerosis and spinal muscular atrophy, by reducing NFL levels, and as such, serve as a therapeutic response biomarker that correlates with clinical efficacy of treatment [260, 261]. Other non-specific biomarkers include, but are not limited to, proteins that delineate synaptic, lysosomal, and mitochondrial functions involved in the formation of intracellular proteins, and participating in the degradation and clearance of abnormally modified proteins or molecules associated with glial activation [25].

Synucleinopathies are associated with impairment of the autophagy-lysosomal pathway which represents a main route for the intracellular degradation of α-syn [262], thus lysosomal activities of the CSF have been a prime area of study for possible diagnosis of synucleinopathies [185, 263, 264]. One such lysosomal enzyme, GCase has been shown to exhibit decreased activity in PD patients compared to healthy controls [185, 265]. Moreover from the BioFIND cohort of 79 PD patients and 61 healthy controls, significant decreases in CSF GCase and cathepsin D activities (-28% and -21%, respectively) were found in PD compared to healthy controls, and a similar trend was also observed for β-hexosaminidase activity (-9% in PD patients) [265]. Moreover, 13% of PD patients and 5% of healthy controls were found to be carriers of mutations of the GCase coding gene (*GBA*). Although GCase activity was lower in carriers compared to non-carriers (-27%), the overall decrease was independent of *GBA* mutation carrier status (-25% in non-carrier PD patients versus non-carrier healthy controls). Receiver Operating Characteristic (ROC) curve analyses showed suboptimal diagnostic accuracies for GCase (sensitivity 67%, specificity 77%) and cathepsin D (sensitivity 61%, specificity 77%). The diagnostic performance improved by combining the panel of all measured lysosomal enzyme activities (sensitivity 71%, specificity 85%), and was further augmented with the inclusion of amyloid, tau, and α-syn pathology biomarkers added to the model [265].

Exosomes can contribute to the pathogenesis of different synucleinopathies as principal mediators of α-syn cell-to-cell transmission. In addition, they can transport RNA, primarily miRNA involved in regulating the expression of different genes associated with PD, DLB, and MSA [266–268]. In several synucleinopathies, α-syn is secreted directly into the extracellular space or transmitted via exosome pathways, and secretion is regulated via intracellular calcium concentrations. Additionally, exosomes containing α-syn are released by damaged neurons to further transmit aberrant α-syn beyond neuronal confines. Thus, exosomes can alter α-syn spread from neuron-to-neuron to neuron-to-glial cell; the latter activates microglia and induces inflammatory foci in areas of the brain [269]. While exosomes carry low levels of α-syn, they were also found to provide an ideal environment for α-syn aggregation, transmission, and synucleinopathy. This is supported by the finding that α-syn oligomers in exosomes are more easily transmitted and accepted by cells than free-form of α-syn species [270]. Exosomes were also found to contribute to non-cell autonomous mediation of neurotoxicity, which further facilitates wide-range transportation of α-syn throughout the CNS and to the peripheral immune system [267, 271]. A potential method of measuring neuron-derived biomarkers of neurodegeneration in the periphery is to measure levels of CNS-specific genes and/or proteins in the exosomes isolated from blood [272]. This offers a less invasive method compared to CSF measurements, however, the sensitivities of some immunoassays, such as ELISA, are not sufficient for quantifying the concentration of CNS biomarkers in blood exosomes [118]. To overcome this problem, advanced and highly sensitive techniques and analytical methods, such as Meso Scale Discovery (MSD) platform, single molecule array (SIMOA), and scRNA-seq, were developed to improve the detection of peripheral biomarkers contained within CNS-derived exosomes [118, 273].

### Therapeutic biomarkers for neurodegenerative diseases

As seen in this review, extensive evidence identified promising diagnostic and prognostic biomarkers for different synucleinopathies that can be measured in CSF or blood. However, the knowledge and identification of therapeutic biomarkers, which track responses to disease treatment, remain enigmatic. For different neurodegenerative disorders, the current approved therapies are palliative for symptomatic relief, and have little or no curative effect on motor and cognitive dysfunctions seen commonly in neurodegenerative disorders. Therefore, an urgent need is warranted for development of DMTs that better control disease progression in neurodegenerative disorders. Identifying therapeutic biomarkers that measure therapy responses and efficacy in blood is an unmet need for clinical studies wherein potential DMTs are evaluated. Our research group, along with others, demonstrated that Tregs attenuate neuroinflammation and protect dopaminergic neurons from injury and loss [178–180]. Our works demonstrated that granulocyte–macrophage colony stimulating factor (GM-CSF, sargramostim, Leukine®) increases Treg numbers and function, protects dopaminergic neurons in PD animal models [178, 274], and improves motor function as determined by UPDRS and magnetoencephalography (MEG)-recorded cortical output in PD patients [180, 181, 275]. Our research group was the first to assess the transcriptomic and proteomic profiles of peripheral blood lymphocytes [180] and monocytes [186] in PD patients treated with an immune modulator drug (sargramostim). Significant increases in IL-10 gene expression by 2 and 6 months after treatment initiation were found, thus supporting the immunosuppressive biomarker expression observed in Treg function. In addition, proteomic analysis indicated that sargramostim treatment downregulates calcineurin and NF-κB expression significantly by 2 months of treatment and their levels remained reduced after 6 months of treatment, suggesting the reduction of inflammation-mediated neurodegeneration and a consequent protective effect in PD patients [180].

In neurodegenerative diseases, the actions of microglia, the resident myeloid cells in the CNS, may diverge from or intersect with those of recruited monocytes to drive immune-mediated pathology [276]. Therefore, we studied the association between monocyte profiles and clinical motor function and disease progression during immune modulatory therapy with sargramostim in PD patients [186]. We showed that monocyte transcriptomic and proteomic signature profiles demonstrate a neuroprotective signature that includes antioxidant, anti-inflammatory, and autophagy genes and proteins (LRRK2, HMOX1, TLR2, TLR8, transcription factor p65; RELA, ATG7, and GABA type A receptor associated protein like 2; GABARAPL2). Our findings showed the predictive potential of LRRK2 gene and protein expression for UPDRS Part III scores and changes in scores. In addition, *HMOX1*, *TLR2*, and *ATG7* gene expression, and RELA, TLR2, and ATG7 protein expression, also showed predictive potential for UPDRS Part III scores and changes in scores, suggesting the utility of these genes/proteins as putative biomarkers for sargramostim therapy. Therefore, these genes/proteins may serve as potential biomarkers to predict therapeutic response in synucleinopathies treated with sargramostim or similar immunomodulatory therapies. Due to the small sample size in that study [186], the therapeutic biomarkers identified are currently being validated in ten PD patients through a twelve-month study in our laboratories (ClinicalTrials.gov Identifier: NCT05677633) [277].

## Conclusions

The need for translational biomarkers for different neurodegenerative diseases is extremely warranted. To uncover potential translational biomarkers, different aspects need be taken into consideration including (1) availability of highly sensitive assays, which are specific to the target biomarker; (2) less invasive sample collection method, such as blood-based samples; (3) mechanisms by which peripheral biomarkers interact with CNS compartments and contribute to disease pathogenesis should be known and well-established [e.g., the intersection between roles of peripheral monocytes and CNS microglia in neurodegenerative disorders]; (4) availability of high-throughput “omics” technologies to investigate the entirety of the genome, proteome, and metabolome for biomarker discovery; (5) availability of up-to-date and well-curated bioinformatics tools for “omics” data analysis; and 6) revelation of drug targets for identification of pharmacodynamic and therapeutic biomarkers, such as Tregs [180] and/or monocytes [186] for sargramostim therapy. Biomarkers for neurodegenerative diseases are needed in the clinic to improve the differential diagnosis, and in drug discovery to facilitate the development and monitoring of effective DMTs.

## Data Availability

Not applicable.

## Abbreviations

^11^C-DTBZ: ^11^C-dihydrotetrabenazine
^123^I-MIBG: ^123^Iodine-meta-iodobenzylguanidine
^18^F-FDG: ^18^F-fluorodeoxyglucose
AAV: Adeno-associated virus
ABCA1: ATP-binding cassette A1
AD: Alzheimer’s disease
APOE: Apolipoprotein E glycoprotein
ATG7: Autophagy related 7
Aβ: Amyloid beta
BIN1: Bridging integrator 1 protein
BBB: Blood–brain barrier
CCL5: C-C motif chemokine ligand 5
CHI3L1: Chitinase-3-like protein 1
CMA: Chaperon mediated autophagy
CNS: Central nervous system
CSF: Cerebrospinal fluid
DAT: Dopamine transporter
DJ-1: Deglycase
DLB: Dementia with LBs
DMTs: Disease-modifying therapies
DTI: Diffusion tensor imaging
ELISA: Enzyme-linked immunosorbent assays
ER: Endoplasmic reticulum
ERAD: ER associated degradation
FTD: Frontotemporal dementia
GBA: Glucocerebrosidase; GCase coding gene
GCase: β-Glucocerebrosidase
GWAS: Genome-wide association studies
HMOX1: Heme oxygenase 1
IFN-γ: Interferon-gamma
IL-1β: Interleukin-1beta
IRBD: Isolated RBD
LBD: Lewy body dementia
LBs: Lewy bodies
LNs: Lewy neurites
LRRK2: Leucine-rich repeat kinase 2
MHC-II: Major histocompatibility complex class II
miRNAs: Micro-ribonucleic acids
MPTP: 1-Methyl-4-phenyl-1,2,3,6-tetrahydropyridine
MRI: Magnetic resonance imaging
MSA: Multiple system atrophy
NBM: Nucleus basalis of Meynert
NFL: Neurofilament light-chain
NF-κB: Nuclear factor kappa-light-chain-enhancer of activated B cells
NGRN: Neurogranin
PD: Parkinson’s disease
PDD: PD dementia
PET: Positron emission tomography
PNS: Peripheral nervous system
PPI: Protein–protein interaction
p-tau: Phosphorylated tau
pTau_181_: P-tau protein at threonine-181
RBC: Red blood cell
RBD: Rapid-eye-movement (REM) sleep behavior disorder
RELA: Transcription factor p65
ROS: Reactive oxygen species
RT-QuIC: Real-time quaking-induced conversion
SAA: Seed amplification assay
scRNA-seq: Single-cell RNA-sequencing
sFIDA: Surface-based fluorescence intensity distribution analysis
*SNCA*: α-Syn gene
*SNCA-AS1*: *SNCA* Antisense RNA 1
SNpc: Substantia nigra pars compacta
SPECT: Single-photon emission computed tomography
Teffs: Effector T cells
TLR: Toll-like receptor
TMEM175: Transmembrane protein 175
TNF-α: Tumor necrosis factor-alpha
Tregs: Regulatory T cells
t-tau: Total tau
UCH-L1: Ubiquitin C-terminal hydrolase-L1
UPDRS: United Parkinson’s Disease Rating scale
UPR: Unfolded protein response
V-ATPase: Vacuolar-type H^+^-ATPase
VILIP-1: Visinin-like protein 1
VMAT2: Vesicular monoamine type 2 transporter
α-syn: α-Synuclein
